# A rapid point-of-care test for vancomycin monitoring at the clinical bedside

**DOI:** 10.1016/j.isci.2026.116064

**Published:** 2026-07-09

**Authors:** Damon T. Burrow, David S. Kinnamon, Jason Liu, Jacob T. Heggestad, Simone Wall, Brooke E. Silverstein, Suhail K. Mithani, Vijay Krishnamoorthy, Richard Drew, Daniel Y. Joh, Angus Hucknall, Ashutosh Chilkoti

**Affiliations:** 1 Department of Biomedical Engineering, Pratt School of Engineering, Duke University, Durham, NC 27708, USA; 2 Division of Plastic, Maxillofacial, & Oral Surgery, Duke University, Durham, NC 27708, USA; 3 Department of Population Health Sciences, Duke University School of Medicine, Durhan, NC 27708, USA; 4 Department of Anesthesiology, Duke University School of Medicine, Durhan, NC 27708, USA; 5 Department of Infectious Diseases, Duke University School of Medicine, Durham, NC 27708, USA

**Keywords:** Vancomycin, therapeutic drug monitoring, point-of-care testing, antibiotic, antibiotic resistance, biosensor, immunoassay

## Abstract

Vancomycin requires individualized dosing guided by therapeutic drug monitoring, but current laboratory assays are slow and centralized. We describe a competition-format microarray assay on a poly(oligo(ethylene glycol) methacrylate-co-glycidyl methacrylate) brush integrated into a single-use microfluidic cassette and portable reader to quantify vancomycin directly from fingerstick volumes of whole blood in 30 min. The assay shows a limit of detection of 0.8 μg/mL and precise quantification across the clinical range, with relative error below 20%. In a pilot study of 31 patient samples, cassette measurements correlated strongly with routine hospital testing with minimal bias, including when operated by minimally trained coordinators. This work establishes an approach for rapid, decentralized vancomycin monitoring that could support area-under-the-curve-based dosing in settings where access to high-complexity laboratories is limited and suggests a path toward extending therapeutic drug monitoring to point-of-care devices.

## Highlights

• A rapid point-of-care test quantifies vancomycin from whole blood within 30 min
• The test showed strong agreement with the gold standard assay in a clinical pilot study
• This work extends the D4 platform beyond proteins to enable therapeutic drug monitoring

## Graphical abstract

**Figure:**
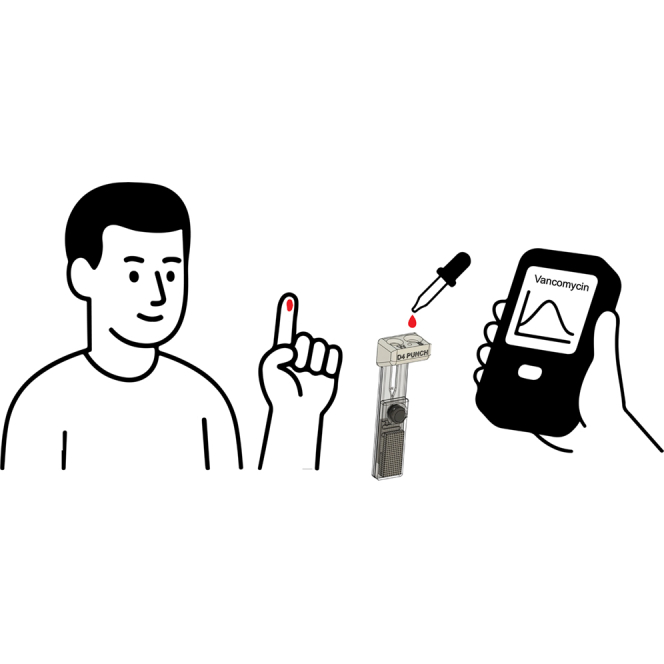

## Teaser

Public health; Medical tests

## Introduction

Vancomycin is an antibiotic used to treat multidrug-resistant Gram-positive bacteria, most notably methicillin-resistant *Staphylococcus aureus* (MRSA).^1^^,^^2^^,^^3^ Vancomycin works by inhibiting cell wall synthesis, which causes bacterial cell death.^1^^,^^4^ Vancomycin has a narrow therapeutic index that results from complex, variable intra- and inter-patient pharmacokinetic and pharmacodynamic differences that often cause initial vancomycin doses to be inadequate.^5^^,^^6^ Overdosing of vancomycin can lead to kidney injury,^7^ while underdosing can contribute to antibiotic resistance.^2^^,^^8^^,^^9^^,^^10^ Precise vancomycin dosing is hence important to balance efficacy while minimizing toxic side effects and the risk of antibiotic resistance.^2^^,^^8^^,^^9^^,^^10^^,^^11^^,^^12^^,^^13^ In 2013, a meta-analysis showed that therapeutic drug monitoring (TDM) of patients on vancomycin had roughly one-fourth the odds of kidney injury compared to those whose concentrations were not monitored.^14^ However, despite its importance, precise dosing of vancomycin is challenging because of its narrow therapeutic index and significant inter- and intra-patient pharmacokinetic and pharmacodynamic differences.^13^^,^^14^

Vancomycin monitoring is currently performed using liquid chromatography-tandem mass spectrometry or immunoassays.^15^^,^^16^^,^^17^^,^^18^ To perform these tests, venous blood samples are taken from patients by phlebotomy, transported to a lab, processed, and then tested with results available in hours to days. The reliance on these relatively inefficient methods increases the risk to patient health due to delayed doses, raises healthcare costs, and limits the geographic reach of VANC TDM practices.^19^^,^^20^ Furthermore, because it is used for so many patients and requires so many tests per patient—for example, each year at Duke University hospital alone, roughly 14,000 VANC tests are performed^21^—improving the efficiency of VANC monitoring would have a transformative effect across healthcare systems.

Because of its clinical importance, best practices for vancomycin monitoring are regularly revisited. Historically, peak (highest) and trough (lowest) measurements have been used to inform adjustments in dose.^22^ However, peak-and-trough method is inadequate, because it captures only isolated snapshots of drug concentration, missing the critical dynamic relationship between drug exposure and therapeutic outcome.^23^ The clinical consequences of improper vancomycin dosing from this oversimplified vancomycin monitoring approach can be severe, with treatment failures resulting in life-threatening infections, emergence of vancomycin-resistant organisms, and preventable kidney damage that prolongs hospital stays and increases mortality.^23^

Updated consensus guidelines in 2020 highlighted the importance of moving beyond a single point-in-time measurement approach to a method where the area under the concentration-time curve (AUC) is used to more precisely map patient drug exposure.^13^^,^^24^^,^^25^^,^^26^^,^^27^^,^^28^ Despite emerging evidence of its clinical utility, the implementation of AUC-based monitoring of vancomycin has seen little progress due to practical constraints. This is because AUC-based monitoring requires multiple measurements throughout the dosing window to accurately calculate the AUC, which significantly increases the number of required tests. This means that without a significant breakthrough in developing and deploying a rapid diagnostic test for vancomycin that can be used at the clinical bedside or at-home, these clinical objectives cannot be realized.

Point-of-care tests (POCTs) have been successfully used for rapid, at-home diagnosis, as seen by the critical role lateral flow assays (LFAs) played during the COVID-19 pandemic.^29^^,^^30^^,^^31^^,^^32^^,^^33^^,^^34^^,^^35^^,^^36^^,^^37^^,^^38^^,^^39^^,^^40^^,^^41^ However, while some POCTs have been reported in the scientific literature for detection of therapeutic drugs, like vancomycin, most have yet to break through into clinical practice.^42^^,^^43^^,^^44^ Sandwich immunoassay-based POCTs that use antibody pairs fail to quantify small molecule and low molecular weight drugs with the necessary accuracy and precision because the small size of many drugs, including vancomycin, limits the opportunity for multiple binders with non-overlapping epitopes to dock simultaneously.^45^ Chromatographic methods, though highly accurate, require extensive sample preparation and sophisticated instrumentation that is unsuitable for bedside use.^46^ Electrochemical sensors show promise but face stability and interference issues in complex biological matrices.^47^ Mass spectrometry remains the gold standard for accuracy but is confined to central laboratories due to size, cost, and technical complexity.^48^ Additionally, unlike other health settings where POCTs have been effectively deployed, such as the pregnancy test that has a binary readout and the blood glucose sensor that operates at high—millimolar—concentrations of glucose, vancomycin requires high precision and accuracy to quantify drug concentrations across a very narrow range from whole blood, which makes it challenging to develop a POCT.

For more than a decade, our group has been developing a POCT platform called the “D4” that can match—and in some cases exceed—the analytical figures of merit of commonly used clinical tests, such as enzyme-linked immunosorbent assays (ELISAs) and LFAs.^49^^,^^50^^,^^51^^,^^52^^,^^53^^,^^54^ The high sensitivity and accuracy of the D4 assay is largely due to the use of a non-fouling “stealth” polymer brush, which significantly reduces background noise.^31^^,^^49^^,^^51^^,^^52^^,^^54^^,^^55^^,^^56^^,^^57^^,^^58^^,^^59^ Additionally, we have developed a microfluidic platform and a fluorescence detector—the D4Scope which is described in detail in previous reports^49^^,^^51^^,^^60^—that converts the D4 assay into a POCT that is compatible with nasopharyngeal swabs, urine, and whole blood.^49^^,^^51^^,^^60^ However, all our previous work with the D4 assay was limited to quantifying protein analytes by a two-antibody sandwich, a scheme that does not work well for low molecular weight analytes, like vancomycin, because it is virtually impossible for two antibodies to bind the small molecule analyte at two different sites because of their small size. In contrast, the present work reports the first D4-based competition assay for a low-molecular-weight therapeutic drug and its integration into a fully portable point-of-care device with initial clinical validation using whole blood.

Motivated by the need for a vancomycin POCT, we explored multiple test formats and we describe herein the successful development of a competition-based VANC-D4 POCT that can quantify vancomycin concentration across its clinically relevant range. Further, we describe results from a two-stage pilot clinical study using whole blood samples from patients at Duke University Medical Center. Importantly, our test demonstrates a strong correlation with the gold standard test, with performance metrics that approach standards set by the Food and Drug Administration for regulatory approval of a clinical diagnostic. Together, this work expands the analytical scope of the D4 platform and establishes a generalizable approach for point-of-care TDM of drugs with a narrow-therapeutic-index.

## Results

### Development of a competition D4 assay for VANC detection

To quantify vancomycin, we developed a competition assay that extends the utility of the D4 assay described in previously.^49^^,^^50^^,^^51^^,^^52^^,^^53^^,^^54^ In pilot studies to identify the best format for vancomycin testing, we explored multiple test formats, such as a competition-based format with an anti-vancomycin antibody used as capture antibody (“cAb” in Figures S1A and S1B) and a competition-based format with anti-vancomycin antibodies used as the detection antibody (“dAb” in Figures S1C and S1D). The dAb competition-based assay demonstrated superior performance to the cAb competition-based assay (Figures S2C and S2D) in correlating with vancomycin measurements provided by the Duke Clinical Automated Laboratory (DCAL). We hence settled upon the dAb competition-based assay format as the best option for vancomycin testing.

Our vancomycin assay utilizes an anti-vancomycin antibody and a BSA-conjugated vancomycin (VANC-BSA) as the competitive antigen. In the assay, the free drug competes with the surface-bound antigen—VANC-BSA—for binding to the antibody. As in previously reported D4 assays, the VANC-D4 assay utilizes a polymer brush on glass that blocks the non-specific binding of proteins in blood and enables the simple immobilization and stabilization of proteins via non-contact inkjet printing. Unlike previous reports, however instead of a poly(oligo[ethylene glycol] methyl ether methacrylate) (POEGMA) brush, we used a copolymer brush made of poly(oligo[ethylene glycol] methyl ether methacrylate-co-glycidyl methacrylate) (POEGMA-co-GMA). This is because POEGMA-co-GMA provided brighter, more consistent microspots that resulted in more accurate and precise vancomycin measurements across its narrow therapeutic window than a POEGMA brush (Figure S4A; Table S1).

**Figure 1:**
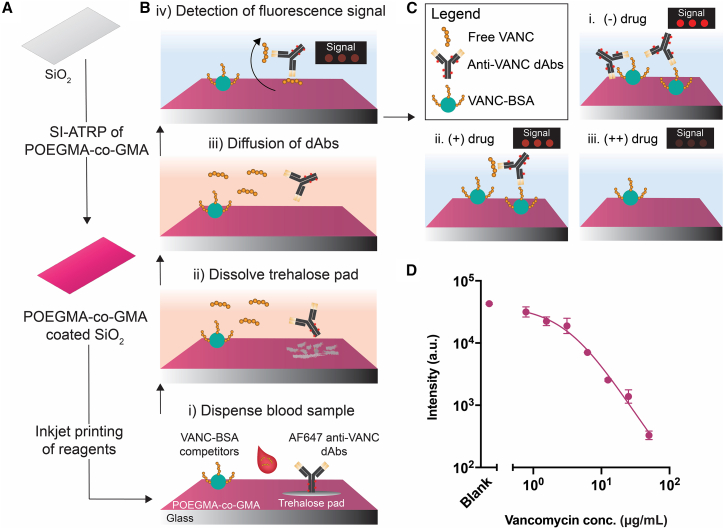
The VANC-D4 assay (A) Fabrication of the VANC-D4 assay. SI-ATRP is used to grow POEGMA-co-GMA brushes on glass slides. Following SI-ATRP, VANC-BSA is inkjet printed into the polymer brush to create stable spots and fluorescently labeled anti-VANC antibodies (termed dAb) are printed on trehalose pads to create labile spots. (B) Sequential steps of the VANC-D4 assay. The steps of the VANC-D4 are (i) dispensing a blood sample on the surface, (ii) dissolution of the trehalose pads to release the fluorescently labeled dAb into solution, (iii) diffusion of the labeled dAb in the sample volume, (iv) detection of fluorescence signal from the antibody bound to immobilized VANC-BSA. (C) Representative VANC-D4 assay response in the presence of (i) no drug, (ii) low drug, and (iii) high drug concentration. (D) VANC monitoring across its clinical range using VANC-spiked whole blood diluted 100-fold in running buffer, analyzed in triplicate. The clinical range for the test is 1–45 μg/mL. Intensity values are shown, with error bars that represent the standard error of the means for *n* = 3 separate runs. The LOD for the presented dose-response curve was calculated to be 0.8 μg/mL.

To grow POEGMA-co-GMA brushes, surface-initiated atom transfer radical polymerization (SI-ATRP) was used (Figure 1A). Following SI-ATRP of a POEGMA-co-GMA brush on glass, competition and detection reagents were inkjet printed on the polymer brush to create the self-contained VANC-D4 assay. First, three layers of trehalose were sequentially printed followed by printing discrete spots of fluorescently labeled anti-vancomycin antibodies on the trehalose brush. Next, VANC-BSA conjugate (with a VANC:BSA molar ratio of ∼7:1) was inkjet printed directly onto the polymer brush to create stable spots. Vancomycin concentrations were measured, following assay runs, by quantifying the fluorescence signal created by Alexa Fluor 647 labeled anti-vancomycin antibodies bound to the stable VANC-BSA spots.

To determine the optimal copolymer composition, a library of copolymer brushes with OEGMA and GMA containing GMA ranging from 1% to 17% of total reaction volume (Figure S3) were fabricated and tested. Results from assays run on this set of slides showed that beyond 5% GMA, the dynamic range of the assay was significantly reduced, so that copolymer composition with greater than 5% GMA were eliminated. In selecting the optimal copolymer, we simultaneously considered (1) relative error across the clinically relevant vancomycin concentration range, (2) the dynamic range of the fluorescence response, and (3) spot-to-spot and run-to-run reproducibility. POEGMA-co-GMA brushes containing 2% GMA provided the best balance of these metrics, with uniformly bright, stable microspots and relative error <20% at all non-anchor points Next, we narrowed down the optimal copolymer composition between 1 and 5% GMA, and found that POEGMA-co-GMA brush coated slides with 2% GMA resulted in the lowest relative error (RE) in quantifying vancomycin. Importantly, the assay run on 2% GMA slides resulted in an RE of <20% (as defined in Equation 1 in the STAR Methods section of this report), which close to the standard defined by the FDA for clinical approval.

The VANC-D4 assay operates through the same sequential steps as other D4 assays (Figure 1B),^49^^,^^51^^,^^54^ where, following sample addition (Figures 1B and 1I), fluorescently labeled detection antibodies (termed dAbs) dissolve from the excipient pads (Figures 1B, ii) and diffuse throughout the sample volume (Figures 1B, iii). After incubation and washing, the dissolved dAbs in solution binds to the immobilized VANC-BSA antigen to produce an inverse fluorescence signal (Figures 1B, iv) that decreases with increasing VANC concentration in the sample, as shown in Figure 1D. The VANC-D4 produces an inverse fluorescence curve because as more vancomycin is present in the system, it increases the fraction of the fluorescently labeled dAb that is bound to the vancomycin in solution, which thereby reduces the amount of free dAb that can bind to the printed VANC-BSA spots. The VANC-D4 assay completes these sequential steps and generates a readout in <30 min.

The VANC-D4 assay is highly sensitive, with a limit of detection (LOD) that is ∼100-fold lower than that required for clinical testing. Tuning the sensitivity of the assay using a dilution buffer enabled us to align the response of the VANC-D4 with vancomycin’s clinical range (Figure S2B). As shown in Figure 1D, the VANC-D4 can produce a dose-response curve that detects vancomycin across its clinically relevant concentration range with fluorescence output spanning over two orders of magnitude in vancomycin concentration.

### Clinical validation of the VANC-D4 assay

Having succeeded in achieving a sensitivity on the VANC-D4 assay with vancomycin spiked into whole blood that exceeded the clinical requirement, we next optimized the VANC-D4 assay to measure vancomycin concentrations across the clinically relevant range of 1%–45 μg/mL. We chose to define this range of concentration as the “clinical range” based on the 5%–20 μg/mL reference range provided by the Duke University hospital’s test catalog^21^ and based on vancomycin concentrations that ranged from <3.5 μg/mL to 37.1 μg/mL in samples that we acquired from Duke University Hospital.

We assessed the precision of the assay by calculating the percent relative error (RE) for all non-anchor points, which are defined as any point between the minimum and maximum points. Anchor points (i.e., minimum and maximum non-blank points) are used to constrain the curve fit and are thus not included for RE analysis. For the curves shown, excluded anchor points are 0.78 μg/mL and 50 μg/mL. Blank values (0 μg/mL) are also excluded from RE analysis as these points are used exclusively to define the LOD of the VANC-D4. The success criteria for precision was defined as an RE <20% for non-anchor points of 25, 12.5, 6.25, 3.125, and 1.56 μg/mL VANC based on recommendations in the FDA’s Bioanalytical Method Validation Guidance for Industry.^61^ Accuracy of the VANC-D4 assay was determined by plotting the correlation between VANC measurements taken on the VANC-D4 assay and reported measurements from the gold standard immunoassay test performed in the DCAL. We defined success for our assay as R^2^ > 0.8.

Through a systematic optimization process, we identified three significant parameters that could be used to tune the performance of the VANC-D4 assay. First, we found that by diluting vancomycin-spiked whole blood samples in a buffer, we could dramatically shift the dynamic range of the assay to align with the concentration regime of vancomycin’s clinical range (Figures S2A and S2C). Second, the amount dAb printed on the cassette can be used to fine-tune the dynamic range of the assay (Figure S4B). Specifically, reducing the amount of dAb improves the accuracy at lower vancomycin concentrations and reduces accuracy at higher vancomycin concentrations, while higher dAb amounts have the opposite effect. This observation is consistent with the format of the assay, as the fluorescence signal arises from the fraction of fluorescently labeled dAbs that are not bound by the free vancomycin in solution and that can bind to the printed VANC-BSA spots on the surface. Third, as discussed previously, we found polymer brushes of POEGMA-co-GMA with 2% GMA resulted in VANC-D4 assays with RE < 20% for non-anchor points, compared to assays fabricated on a pure POEGMA brush that had an RE > 20% (Figure S4A). We hypothesize that incorporation of a small fraction of GMA into the POEGMA brush introduces epoxy groups that can react with lysine residues on the VANC-BSA conjugate, increasing both the density and covalent stabilization of immobilized antigen. At the same time, the underlying POEGMA component preserves the non-fouling characteristics of the brush. This combination yields microspots that are brighter and more uniform across the slide and between batches, thereby reducing variability in fluorescence readout of the assay, thereby enabling more precise vancomycin quantification within its narrow therapeutic window. These hypotheses are supported by previous reports by Hu et al. and others.^62^^,^^63^^,^^64^

**Figure 2:**
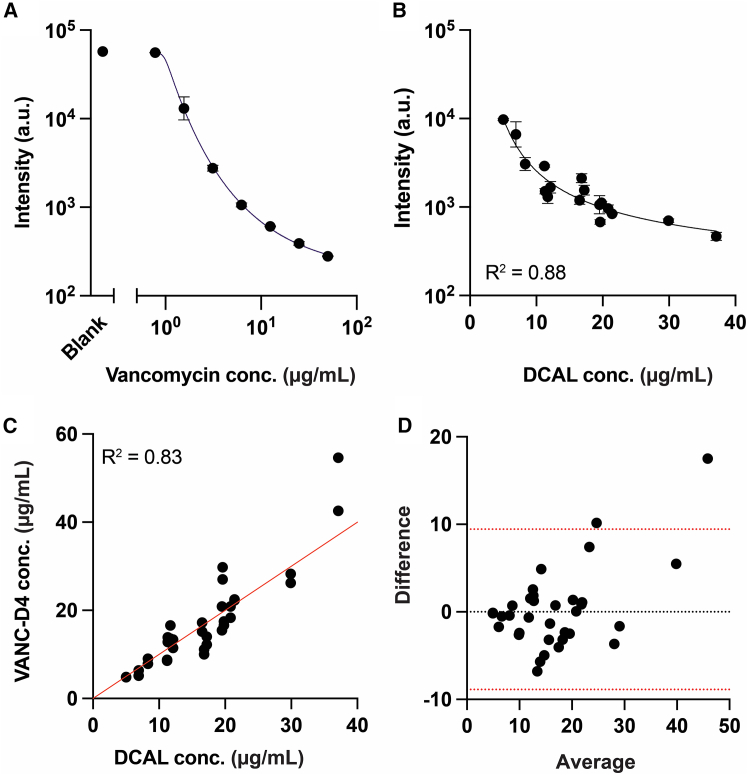
Clinical comparison of the VANC-D4 assay to assay carried out by DCAL (A) Optimized VANC-D4 dose response calibration curve. Data are *n* = 2, plotted as mean ± SD. (B) VANC-D4 fluorescence response curve plotting the log of the VANC-D4 fluorescence (*y* axis) and measured VANC values from DCAL (*x* axis). R^2^ value is defined using the same asymmetric sigmoidal 5 parameter curve fit as Figure 2A. Data are represented as the mean of *n* = 2 measurements ± SD. (C) Correlation plot of VANC values as measured by the VANC-D4 (*y* axis) and DCAL (*x* axis). In this plot, the red line is the line of identity. (D) Bland-Altman analysis plot comparing the VANC-D4 and DCAL immunoassay. Sample number = 34 (17 patients run in duplicate). Mean difference (bias) = 0.2845 μg/mL. Standard deviation = 4.672 μg/mL 95% limits of agreement (red lines), lower limit = −8.873 μg/mL and upper limit = 9.442 μg/mL.

Figure 2 shows the performance of the optimized VANC-D4 assay fabricated on POEGMA-co-GMA brushes with 2% GMA, printed dAb at 0.05 mg/mL, and a 100-fold dilution of whole blood samples. The optimization of these three parameters yielded a VANC-D4 assay that displays the expected inverse fluorescence dose-response behavior in both VANC-spiked samples (Figure 2A) and in clinical samples with known vancomycin values that were independently measured by DCAL and provided to us (Figure 2B). Critically, the optimized VANC-D4 assay shows an RE < 20% for all non-anchor points and excellent correlation with the gold standard test independently performed at DCAL with an R^2^ of 0.83 from 17 individual patient samples, all run in duplicate, plotted as single values on the graph in Figure 2C. For this test, a correction factor of 2.8 was applied to the raw measurement values from the VANC-D4 to align the measurements with the values from DCAL. To further characterize the performance of our assay against the gold standard, we performed a Bland-Altman analysis (Figure 2D). This analysis showed a very low systematic bias of 0.2845 μg/mL between the VANC-D4 and the DCAL test.

### Translating the VANC-D4 to a POCT

The data presented thus far were generated using VANC-D4 assays run in an open-format (Figure S6). In this format, up to 24 assays can be run in parallel on a single slide in a single experiment, similar to an ELISA. The open-format is useful for high-throughput optimization of antibody print conditions and assay protocols but does require manual timing and wash and drying steps to complete the assay. Additionally, open-format assays require the use of centrifuges and a benchtop commercial Genepix scanner to obtain results.

While valuable for optimization, the open-format is not amenable to the bedside and eventual in-home workflows that are the ultimate objectives of our test. Following completion of our optimization studies, we adapted the VANC-D4 assay into a fully deployable POCT. To achieve this, we built upon our previous work translating sandwich immunoassays into microfluidic POCTs.^51^^,^^60^^,^^65^ However, the competitive assay format employed by the VANC-D4 assay posed unique challenges, requiring the redesign of the microfluidic cassette and the sample-handling workflow.

First, we implemented an off-cassette pre-mixing strategy, where the sample is combined with dAbs in a buffer consisting of phosphate buffered saline (PBS) and 1% Triton X-100 prior to introduction onto the test. This provides two crucial functions: (1) it achieves the required sample dilution to align the assay’s dynamic range with clinically relevant VANC concentrations, and (2) it reduces assay variability by ensuring uniform vancomycin-dAb interactions prior to sample addition to the cassette. Unlike the open-format D4 assay, where a plate rocker is used to facilitate dAb dissolution and mixing, the microfluidic cassette relies on convective forces—introduced upon sample loading—and diffusion to dissolve and deliver dAbs. We demonstrate that this pre-mixing step significantly enhances the performance of the competitive assay when compared to direct sample application to the POCT (Figure S6A). Importantly, because dilution is already required to achieve detection within the clinically relevant range of vancomycin concentrations, adding the dAb to the dilution buffer does not introduce additional complexity to the assay workflow.

**Figure 3:**
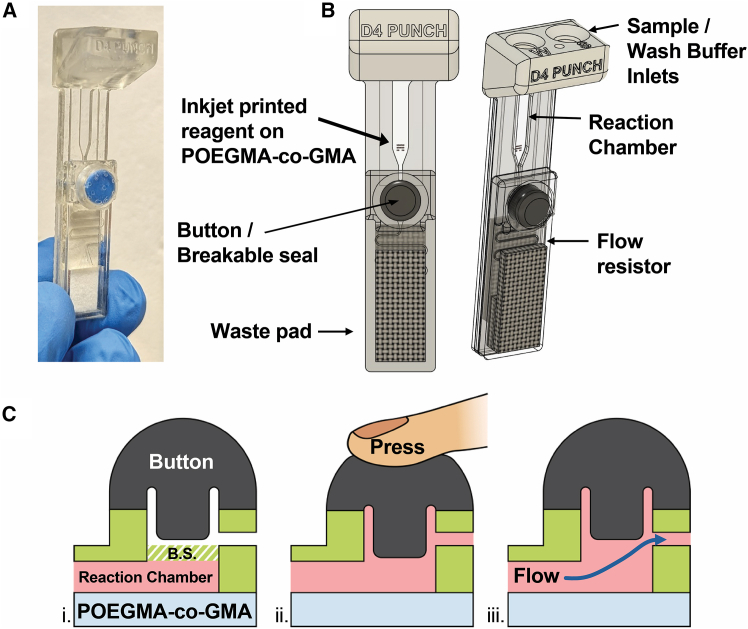
The Punch cassette (A) Photograph of the “punch” cassette. (B) 3D CAD renderings of the cassette with key features identified. (C) Cross-sectional schematic detailing how the punch mechanism functions. (i) The sample incubates for a user-specified time in the reaction chamber upstream from the breakable seal (B.S.). (ii) The user presses the button breaking the seal between the reaction chamber and the downstream flow resistor. (iii) Sample and upstream wash buffer is now free to traverse through the flow resistor and into the waste pad.

Second, we designed a “punch” cassette (Figure 3) that incorporates several improvements over previous designs.^51^^,^^60^^,^^65^ In earlier iterations, following the addition of a sample and wash buffer, a small volume of fluid continuously moved through a microfluidic channel network driven solely by capillary and gravitational forces. These channels controlled both the assay incubation time and the wash phase.^51^^,^^60^^,^^65^ In our redesigned cassette, the capillary-driven network has been replaced with a manually operated breakable seal. This seal keeps the reaction chamber isolated and releases the sample and upstream wash buffer into a downstream waste area only after the desired incubation time has elapsed. In contrast to prior iterations where incubation timing was dictated by channel microarchitecture, the punch cassette is capable of accommodating virtually any incubation time and sample type without the need for test-specific modification. Furthermore, this precisely controlled timing results reduced batch-to-batch and run-to-run variability, which is critical for the VANC-D4 assay.

**Figure 4:**
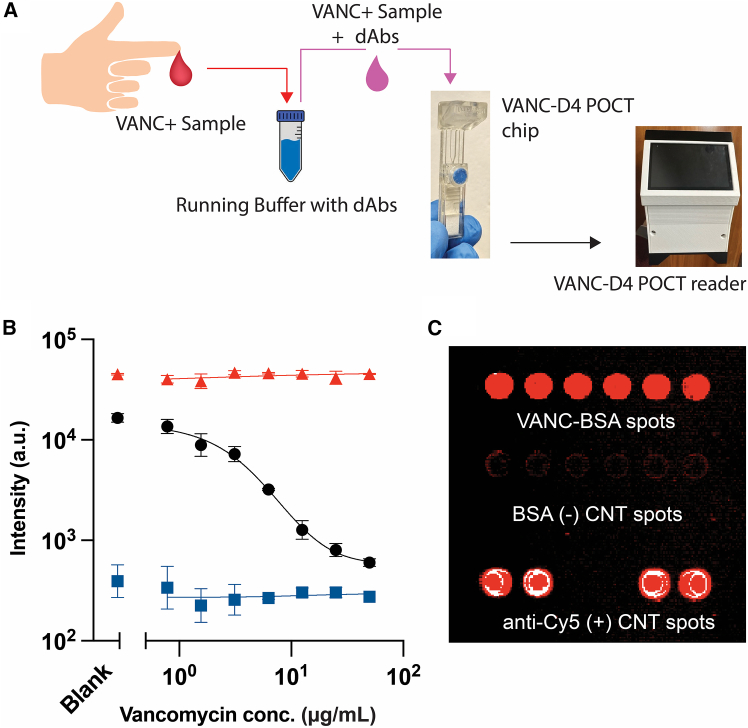
The VANC-D4 POCT (A) Steps to perform the VANC-D4 POCT. Step 1: 20 μL blood is transferred to a tube containing 1980 μL running buffer and anti-vancomycin dAbs. Step 2: user gently rocks tube to homogenize samples and begin anti-vancomycin dAb–vancomycin reaction. Step 3: 80 μL of the diluted sample is loaded on to the VANC-D4 POCT punch cassette. Step 4: incubation until the user presses the button on the VANC-D4 POCT cassette. Step 5: user loads cassette into the VANC-D4 POCT reader cassette chamber (see Figure 6A below for a more detailed view of the cassette chamber) to begin automated analysis. (B) Dose-response curve (black line) of the VANC-D4 POCT with BSA negative (blue line) and anti-Cy5 positive (red line) controls. Data are represented as mean ± SD (*n* = 2 for all lines). (C) GenePix image of VANC-BSA, BSA (−), and anti-Cy5 (+) microspots on the VANC-D4 POCT 3.125 μg/mL vancomycin spiked into whole blood.

**Figure 5:**
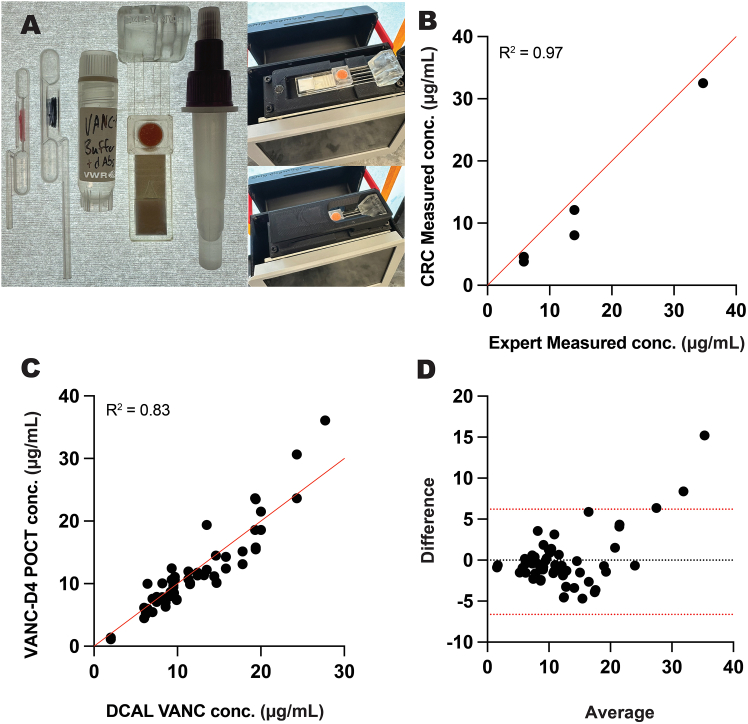
Clinical validation of the VANC-D4 POCT (A) The VANC-D4 POCT “kit”, including disposable pipettes and pre-made reagent tubes. Contents of the kit are as follows from left to right: (1) 20 μL disposable pipette used for transferring blood sample into running buffer with dAbs, (2) 80 μL disposable pipette used to transfer mix of blood, buffer, and dAbs into the VANC-D4 POCT cassette, (3) VWR tube used to store running buffer + dAbs, (4) VANC-D4 POCT punch cassette, (5) Disposable dropper containing 600 μL of wash buffer. Top right image: VANC-D4 POCT cassette loaded in the D4Scope and bottom right image: VANC-D4 POCT secured and ready for imaging. (B) Correlation plot comparing the performance of the VANC-D4 POCT in the hands of minimally trained CRC users (*y* axis) and expert users in our lab (*x* axis). (C) Correlation plot of vancomycin positive clinical samples measured on the VANC-D4 POCT (*y* axis) and at DCAL (*x* axis). (D) Bland-Altman analysis plot comparing the VANC-D4 POCT and DCAL immunoassay. Sample number = 62. Mean difference (bias) = −0.2056 μg/mL. Standard deviation of bias = 3.275 μg/mL 95% limits of agreement (red lines) = −6.625 to 6.214 μg/mL.

The operation of the punch cassette is illustrated in Figure 3C. Upon introduction of the sample and wash buffer into their respective inlets at the start of a test, incubation in the reaction chamber proceeds undisturbed until the seal is broken. After the desired incubation time has elapsed, the user presses a button that breaks the seal, connecting the reaction chamber to a downstream flow resistor and waste pad. The flow resistor facilitates the rapid but controlled removal of sample from the reaction chamber, followed by the flushing of the reaction chamber with the upstream wash buffer. The waste pad then collects the sample and washes fluids. This pad can also be impregnated with decontaminating salts to ensure the cassettes are safe to handle after use. This automated wash process quickly and efficiently removes unbound analyte and fluorescently labeled antibodies from the microarray surface, utilizing surface tension to dry the assay surface prior to imaging with the portable D4Scope shown in Figures 4A v, 5A vi, and vii.

The workflow for the VANC-D4 POCT, enabled by the sample pre-mixing strategy and punch cassette design, is outlined in Figure 4A. First, patient blood from a fingerstick or venous draw is added to a tube containing running buffer with dAbs. For our current iteration of the test, 20 μL of blood was used, as this was the smallest volume compatible with the exact volume transfer pipettes that leveraged in our workflow. While smaller volumes are theoretically possible, they are more prone to subtle errors that negatively impact test performance. Mixing the blood with 1.98 mL of the buffer achieves the 100-fold dilution necessary to accurately quantify vancomycin concentrations. Next, a 80 μL exact volume transfer pipette is used to transfer the diluted blood and dAb mixture onto the VANC-D4 POCT cassette. The cassette is incubated for 30 min, after which the button is pressed to release the sample and upstream wash buffer. Once all liquid drains into the waste pad, the cassette is ready for imaging. A 30-min incubation was empirically determined to be the minimum time necessary to achieve the precision and accuracy thresholds for vancomycin quantification. With continued refinement, future versions of the test may be capable of readout in less than 30 min.

Figure 4B shows a dose-response curve created using the integrated strategy of pre-mixing and precise microfluidic timing summarized in Figure 4A. Critically, we achieved analytical performance comparable to our open-format VANC-D4 assay while realizing the simplicity necessary for point-of-care use. To further improve robustness and user experience, we have also built in on-cassette positive and negative “self-check” controls. Stable fluorescence responses for these controls appear as red (positive) and blue (negative) lines in Figure 4B and are shown in representative images in Figure 4C. These controls serve as essential indicators for identifying cassette failures or workflow errors at the time of data acquisition and analysis.

### Proof-of-concept VANC-D4 POCT clinical testing at Duke University Medical Center

Following successful laboratory validation of the VANC-D4 POCT using vancomycin-spiked donor blood, we conducted a two-stage clinical proof-of-concept study. Stage 1 aimed to validate the VANC-D4 POCT’s accuracy and precision against the gold standard test at DCAL, like before in our open-format tests, while stage 2 aimed to evaluate the test’s usability by health care personnel at DUMC in a simulated clinical setting. Figure 5A below shows the VANC-D4 POCT “kit” that was used for all proof-of-concept tests at DUMC.

In stage 1, we analyzed 26 whole blood samples with known vancomycin concentration from DCAL. The vancomycin concentrations as measured by DCAL and those measured by the VANC-D4 POCT are shown in Table S2. The deidentified patient demographics (race, age, and sex) for each sample can are in Table S2. For each sample, measurements from DCAL were provided as a single measurement for each sample (*n* = 1) as this is how DCAL normally tests samples and reports values. On the VANC-D4 POCT, we tested each sample twice (*n* = 2) to determine both accuracy—how well VANC-D4 POCT reported measurements agreed with DCAL measurements—and precision—how well run 1 and run 2 for each sample agreed with each other. For these tests, investigators were blinded to the measurements from DCAL until after samples were quantified by the VANC-D4 POCT. In these samples, the VANC-D4 POCT demonstrated a strong correlation (R^2^ = 0.83) with DCAL measurements for vancomycin concentrations spanning from <3.5 to 27.7 μg/mL with no significant outliers.

Stage 2 of the clinical pilot study evaluated the performance of the VANC-D4 POCT operated by Clinical Research Coordinators (CRCs) in DUMC’s infectious disease division to assess the test’s robustness and ease of use by minimally trained users. Before testing clinical specimens, we first validated our point-of-care workflow and the VANC-D4 POCT kit by comparing vancomycin-spiked blood samples measured on tests operated by experts in our lab versus minimally trained CRC operators (Figure 5B). Unlike laboratory testing, the CRC-led workflow implemented at DUMC used disposable pipettes (instead of laboratory-grade mechanical pipettes) and a pre-allocated dAb-running buffer mix (rather than fresh dAb aliquots made immediately before testing). Following workflow validation, five vancomycin-positive patient samples acquired at DUMC with their vancomycin concentration independently measured by DCAL were tested by CRCs. Again, the VANC-D4 POCT showed excellent agreement (R^2^ = 0.83 for only CRC-led tests) with measurements taken at DCAL.

Figure 5C shows the correlation between vancomycin concentration measured on the VANC-D4 POCT and DCAL measured concentrations. This plot includes both tests run by members of our group and by CRCs with an aggregate R^2^ value of 0.83. All samples were tested in duplicate, generating 61 successful measurements of our attempted 62 tests (98% success rate). One measurement (patient 2, sample run 2 for the CRC-led tests) was rejected due to a cassette failure. To further compare the results of the VANC-D4 POCT and measurements from DCAL, we performed a Bland-Altman analysis shown in Figure 5D. Overall, the VANC-D4 POCT demonstrated good performance with virtually no bias (−0.2056 μg/mL).

## Discussion

Efficient TDM for vancomycin and other drugs improves patient health outcomes and quality of life while reducing healthcare costs and enabling emerging clinical best practices.^66^^,^^67^ Vancomycin is one of more than 250 narrow therapeutic index drugs whose therapies would benefit from unlocking efficiency gains with a POCT.^68^ We believe new POCTs are essential to meet current and future TDM demands, especially for AUC-based monitoring of NTI drugs, particularly vancomycin. This demand is illustrated by a recent meta-analysis, which found that AUC-guided vancomycin dosing reduces nephrotoxicity by 37.5% (more for select subgroups), compared to trough-based monitoring, while maintaining equivalent clinical efficacy.^67^

The VANC-D4 POCT has the potential to transform current clinical workflows by supplementing existing laboratory tests to enable rapid, near-patient measurements of drug concentrations in decentralized settings. Rapid turnaround is particularly valuable in critical care settings where quick therapeutic decisions can significantly impact patient outcomes and in clinics where on-site testing is unavailable, such as outpatient clinics and rural hospitals.^69^ Instead of the typical scenario in which clinicians may wait hours after initiating vancomycin therapy to obtain the first concentration, a ∼30-min point-of-care result could allow earlier dose adjustments in outpatient infusion centers, smaller hospitals without on-site laboratories, intensive care units, or hospital wards of larger tertiary-care hospitals where samples are currently batched and transported to a central laboratory for analysis. However, deployment in these settings would need to adhere to the infection-prevention and blood-handling policies of each institution and comply with national and international regulatory requirements. In-home use should be viewed as a potential future application that would require dedicated human-factors studies, careful evaluation of infection-control considerations, and appropriate regulatory oversight before implementation.

Importantly, our prototype VANC-D4 POCT exceeds the performance of previously reported POCTs for vancomycin TDM and approaches FDA standards.^43^^,^^70^^,^^71^ The test demonstrates reliable operation by minimally trained users at DUMC, delivering results in 30 min. Going forward, we plan to further investigate the clinical efficacy of our POCT by testing the VANC-D4 POCT in outpatient settings where central laboratory testing is unavailable or logistically challenging.^72^ This expansion will include a comprehensive user study with 10+ participants across different healthcare roles (nurses, lab technicians, and medical staff) to evaluate ease of use, reliability, and workflow integration based on criteria set by the FDA.^61^ The user study will assess the seven key areas outlined in the VANC-D4 POCT User Survey (Appendix A): participant demographics, training effectiveness, procedural performance, user confidence, device assessment, implementation feedback, and observer assessment. Preliminary VANC-D4 POCT user survey results from a CRC tester are shown in Figure S8.

Looking beyond vancomycin, the D4-POCT platform technology has significant potential for broader clinical impact. Vancomycin represents just one of over 250 narrow therapeutic index drugs requiring frequent TDM, including critical medications like aminoglycosides, anticonvulsants, and immunosuppressants.^68^ We have already begun developing similar tests for antibiotics like gentamicin and immunosuppressants such as tacrolimus and sirolimus. The ability to rapidly measure multiple drug concentrations could revolutionize therapeutic monitoring in transplant patients, who often require careful balancing of immunosuppressive medications.^73^^,^^74^^,^^75^^,^^76^^,^^77^ Furthermore, the platform’s potential for multiplexed detection could enable simultaneous monitoring of drug concentrations, organ function, and disease markers, providing a more comprehensive view of patient status in a single test.^78^^,^^79^^,^^80^

### Limitations of the study

While promising, this study and the VANC-D4 POCT have limitations. First, our clinical validation was intentionally limited in scope: we evaluated 31 samples from a single academic medical center, which is unlikely to fully capture the diversity of patient populations, co-medication regimens, and practice patterns in which this test might ultimately be deployed. As such, our data should be interpreted as a proof-of-concept demonstration of analytical performance and preliminary clinical concordance, rather than as a definitive multi-center trial. Future work will include larger-scale validation across multiple healthcare centers and care environments—including outpatient and resource-limited settings—to assess the robustness of the VANC-D4 POCT across different demographics, disease states, and workflows.

Second, while our 30-min turnaround time represents a significant improvement over centralized laboratory testing, further optimization of the pre-mixing and incubation steps could potentially reduce this time to better serve acute care and in-home settings. Potential strategies to shorten assay time include redesigning the microfluidic channel architecture to enhance convective transport and reduce diffusion distances, tuning detection antibody concentration and binding kinetics to accelerate signal formation and exploring abbreviated incubation protocols combined with calibration models that compensate for partial equilibrium. We anticipate that, depending on the clinical use case, future versions of the VANC-D4 POCT could be tailored to trade-off between assay time, sensitivity, and operational simplicity.

Third, the current requirement for pre-mixing of samples introduces a potential source of user error that could affect test accuracy. Notably, although we implemented pre-mixing as an off-cassette step in this prototype, the underlying chemistry does not require manual mixing. In future iterations, this step could be automated by integrating pre-loaded dried reagents and buffer within the cassette, such that the user only introduces a defined volume of blood into a closed cartridge. This design is expected to further reduce user-to-user variability while preserving the assay conditions that yield accurate and precise vancomycin measurements, though it introduces engineering challenges that were out of scope for this study. Additionally, while the test’s performance in non-standard operating conditions—including varying temperature and humidity levels, and extended reagent storage—has been evaluated for other D4 assays,^54^^,^^60^ it remains to be systematically characterized for the VANC-D4 POCT. In future studies, we will perform accelerated stability and real-world environmental testing to quantify assay performance and reagent shelf life across a range of storage and operating conditions (e.g., refrigerated, room temperature, and elevated temperature/humidity). These studies will be essential to support eventual clinical deployment and regulatory submission of the VANC-D4-POCT.

## Resource availability

### Lead contact

Further information and requests for resources and reagents should be directed to and will be fulfilled by Dr. Ashutosh Chilkoti, PhD, email: chilkoti@duke.edu.

### Materials availability

This study did not generate new unique reagents.

### Data and code availability

• All data reported in this paper will be shared by the lead contact upon request.
• This paper does not report original code.
• This paper did not generate new unique reagents.

## Acknowledgments

We thank M. Atkinson for IRB assistance, K. Frankey and E. Howington at CHTN for assistance in acquiring vancomycin positive samples and corresponding DCAL measurements, and CRCs S. Li and C. Daly for their help in acquiring and testing vancomycin samples at DUMC. We also thank M. Datto and S. McCall at DCAL for their helpful guidance. We acknowledge the Shared Materials Instrumentation Facility (SMIF), a member of the North Carolina Research Triangle Nanotechnology Network (RTNN), which is supported by the 10.13039/100000001National Science Foundation (award number ECCS-2025064) as part of the National Nanotechnology Coordinated Infrastructure (NNCI) for facilitating characterization by XPS in this study. This project was supported in part by funding from the 10.13039/100000002National Institutes of Health (no.: 1R01AI150888-01A1).

## Author contributions

Conceptualization, D.T.B., D.S.K., J.T.H., D.Y.J., and A.C.; investigation, D.T.B., D.S.K., J.L., J.T.H., S.W., and B.E.S.; project administration, D.T.B., A.C., D.Y.J., S.K.M., V.K., and R.D.; supervision, A.C., A.H., and S.K.M.; writing – original draft, D.T.B. and D.S.K.; writing–review and editing, D.S.K., J.T.H., and A.C.

## Declaration of interests

The technology described herein has been licensed by SimplusDx from Duke University. A.C., D.S.K., J.T.H., and A.H. are cofounders of SimplusDx and have equity in the company. The following patents are related to this work: granted: 8796184, 9482664, 11169150, 12031912, and 18042032, provisional: 63/839582, patent pending: PCT/US2025/040378, and a provisional patent pending titled: METHOD FOR QUANTIFYING SMALL MOLECULE DRUGS ON A POINT-OF-CARE TEST.

## STAR★Methods

### Key resources table

**Table:** 

REAGENT or RESOURCE	SOURCE	IDENTIFIER
**Antibodies**		

RHA™ anti-vancomycin monoclonal antibody	Creative Diagnostics	Catalog Number: HMABPY054

**Chemicals, peptides, and recombinant proteins**		

Vancomycin [BSA]	Creative Diagnostics	Catalog Number: DAG3035

**Other**		

Vancomycin hydrochloride, Molecular Biology Grade	ThermoFisher	Catalog Number: J62790.06

### Experimental model and study participant details

#### Human study participants

This study involved human participants from whom residual or prospectively collected whole blood samples were obtained for vancomycin testing. All research was conducted under a protocol reviewed and approved by the Duke University Health System Institutional Review Board (IRB Protocol Pro00109699, Principal Investigator: Ashutosh Chilkoti). Informed consent was obtained from all prospectively enrolled participants. De-identified residual samples were acquired through the Cooperative Human Tissue Network (CHTN) under existing CHTN consenting frameworks.

Participants were adult patients undergoing routine vancomycin therapeutic drug monitoring at Duke University Medical Center (DUMC). De-identified demographic information—including age, sex, race, and ethnicity—for each sample was provided by CHTN and is described, when known, in Table S2. A diverse donor pool was used for assay development: more than 100 whole blood samples purchased from Innovative Research (catalog # IWB1K2E10ML) spanning diverse ethnic and age demographics were used; an index of known donor information is provided in Table S3. Because this study used residual clinical samples and was not designed to test hypotheses about the influence of sex, gender, ancestry, race, or ethnicity on assay performance, sex- and gender-based subgroup analyses were not conducted; this represents a limitation of the current work and will be addressed in future larger-scale validation studies.

Sample size was not determined by formal power calculation for this pilot validation study. In Stage 1, 62 de-identified remnant samples with known vancomycin concentrations were obtained through CHTN. In Stage 2, five additional samples were collected prospectively by Clinical Research Coordinators (CRCs) at DUMC. Investigators were blinded to DCAL-measured concentrations until after VANC-D4 POCT measurements were recorded.

### Method details

#### Study design

The goal of this study was to develop a POCT for vancomycin that requires urgent and frequent TDM. The development process comprised three key phases: (1) establishing low molecular weight detection capabilities using the D4 platform, (2) adapting a successful vancomycin assay to a point-of-care workflow, and (3) validating POCT performance against gold standard laboratory methods while maintaining speed and cost advantages. All commercial reagents (drugs, drug conjugates, antibodies) and human whole blood were obtained from commercial suppliers. Initial assay development and optimization utilized vancomycin-spiked whole blood from multiple donors. Clinical validation proceeded in two stages: first using de-identified remnant samples from vancomycin-monitored patients obtained through the Cooperative Human Tissue Network (CHTN), followed by prospectively collected samples obtained by CRCs at Duke University Medical Center. The protocol was reviewed and approved by the Duke University Health System Institutional Review Board. Investigators were blinded to sample concentrations except where explicitly noted in the pilot experiments.

#### VANC-D4 Assay Fabrication

Fabrication of the VANC-D4 assay followed previously published methods for traditional D4 assays^49^^,^^52^^,^^54^ apart from two significant deviations: first, the VANC-D4 is built upon POEGMA-co-GMA brushes in place of POEGMA-only brushes; and second, the VANC-D4 uses VANC-BSA (Creative Diagnostics Catalog Number: DAG3035) in place of capture antibodies. Polymerization of POEGMA-co-GMA brushes with 2% GMA was accomplished using the following method: silicon oxide glass microscope slides were first initialized with a amine group via submersion overnight in (3-aminopropyl)triethoxysilane (IUPAC: 1-propanamine,3-(triethoxysilyl), Sigma-Aldrich Catalog Number: 440140, CAS Number: 919-30-2), followed by functionalization using triethylamine (IUPAC: N,N-diethylethanamine, Sigma-Aldrich, Catalog Number: 471283, CAS Number: 121-44-8) and bromoisobutyryl bromide (IUPAC: 2-bromo-2-methylpropanoyl bromide, Sigma-Aldrich, Catalog Number: 252271, CAS Number: 20769-85-1), then immersed in a pre-mixed polymerization solution containing 330 mL of DI water, 500 mL methanol, 150 mL poly(ethylene glycol) methyl ether methacrylate (PEGMEM) monomer (IUPAC: 2-methoxyethyl methacrylate, Mn 300 average ethylene glycol repeat length 5, Sigma-Aldrich Catalog Number: 447935, CAS Number: 26915-72-0) 20 mL glycidyl methacrylate (GMA) monomer (IUPAC: (oxiran-2-yl)methyl 2-methylprop-2-enoate, Sigma-Aldrich Catalog Number: 779342, CAS Number: 106-91-2), 130 μL hexamethyltriethylenetetramine (HMTETA) (IUPAC: N’-[2-[2-(dimethylamino)ethyl-methylamino]ethyl]-N,N,N′-trimethylethane-1,2-diamine, Sigma-Aldrich Catalog Number: 366404, CAS Number: 3083-10-1), and 77 mg Cu(Ⅱ)Br (ThermoFisher Scientific, Catalog Number: 206331000, CAS Number: 7789-45-9). Surface-initiated atom transfer radical polymerization (SI-ATRP) was carried out in a mBRAUN MB10 LABstar glove box (Serial Number 13–210) for 2 h. Successful polymerization of POEGMA-co-GMA brushes was confirmed by measurement of the polymer brush thickness in air using a J.A. Woollam alpha 2.0 spectroscopic ellipsometer, and by X-ray photoelectron spectroscopy (XPS) on a ThermoFisher Scientific Nexsa G2 surface analyzer (Catalog Number: 9961253).

Following polymerization, D4 assays were fabricated using non-contact inkjet printing methods as described elsewhere.^49^^,^^54^ VANC-BSA was printed using a non-contact inkjet microarray printer (Scienion, sciFLEXARRAYER Type S12). VANC-BSA conjugates were printed from a stock concentration of 1 mg/mL of VANC-BSA. For open-format VANC-D4 assays, anti-vancomycin antibodies (Creative Diagnostics catalog # HMABPY054) were first labeled with Alexa Fluor 647 antibody labeling kits (ThermoFisher Scientific catalog # A20173) following the manufacturer’s protocol, then were printed at 0.025–0.05 mg/mL using a BioDot AD1520 Aspirate Dispense System. For dAb-spike experiments, unless explicitly noted, dAbs for the VANC-D4 POCT were spiked at a concentration of 2.5 μg/mL. For vancomycin-spiked assays, Molecular Biology Grade vancomycin hydrochloride (ThermoFisher Scientific, catalog #J62790.06) was serially diluted into donated human whole blood purchased from Innovative Research (catalog # IWB1K2E10ML). For the studies described here, more than 100 blood samples from different individual donors representing diverse ethnic and age demographics, were used. A complete index of known information for all donors in this study is provided in Table S2.

As described above, for VANC-D4 assays, on-cassette quality controls were added to troubleshoot assays during development and as “self-check” controls in VANC-D4 POCTs. We printed two types of quality control spots on the cassettes to validate each test: 1) anti-Cy5 antibody spots (positive control) that specifically bind to Alexa Fluor 647 labels on the detection antibodies, generating a strong signal only when detection antibodies are incorporated in the pre-mixing step; and 2) unconjugated BSA (negative control), which should exhibit minimal signal due to the high specificity of anti-vancomycin antibodies. An elevated signal at negative control spots indicates unwanted non-specific binding that could compromise assay performance. This dual-control system provides immediate visual verification of proper test execution, with positive controls appearing consistently bright and negative controls remaining dim across all vancomycin concentrations. This approach enables rapid identification of procedural errors or problems with the reagents.

#### Punch cassette fabrication, assembly, and operation

The microfluidic punch cassettes were fabricated using a multilayer approach combining CLAREX acrylic sheets of varying thickness (0.2, 0.5, 1, and 2 mm; Astra Products), pressure sensitive adhesive tapes (7955 MP and 468 MP; 3M Company), and cotton absorbent liner (Whatman CF7; Cytiva Life Sciences). Component geometries were designed in AutoCAD 2024 (Autodesk, Inc.) and precision-cut using a Gravograph LS900 CO2 laser system with Gravostyle 8.0 software. 3D printed accessories were designed in Fusion 360. Fluid reservoirs and button covers were fabricated using a Form 4 stereolithography printer (Formlabs, Inc.) using Clear V5 and Elastic 50A V2 resins. The pin used to break the seal was fabricated using a Bambu X1C using standard PLA filament.

Printing of reagents was the same as described in “VANC-D4 assay fabrication” with the exception being that dAbs for the VANC-D4 POCT were spiked and not printed on the cassette. The VANC-D4 POCT workflow consists of five simple steps (Figure 5A). *First*, the user mixes 20 μL of whole blood with 1.98 mL of running buffer containing anti-vancomycin antibodies (2.5 μg/mL) and detergent (1% Triton X-100, 1 M Tris, 500 mM NaCl, 40 mM EDTA in PBS). *Second*, after gentle mixing to initiate the reaction, 80 μL of this mixture is loaded into the sample inlet of the microfluidic cassette. *Third*, the user then adds 600 μL of wash buffer to the dedicated wash reservoir and allows the test to incubate for 30 min. *Fourth*, following incubation, pressing the cassette’s button initiates an automated wash sequence. *Fifth*, the cassette is inserted into the D4Scope for automated image capture and analysis.

#### VANC-D4 assay performance characterization

Initial assay performance was evaluated by calculating a LOD using established methods.^81^ Success was defined as a VANC-D4 assay with a LOD of less than 1 μg/mL, based on vancomycin reference range.^21^ A VANC-D4 run using undiluted vancomycin-spiked whole blood is shown in Figures S2A and S2C. Assay performance was further evaluated through systematic characterization of precision and accuracy across vancomycin’s clinical range (1–40 μg/mL). Precision was measured using relative error (RE) calculated by the following formula:

(Equation 1) $$RE=absolute\;value\left(\left(\frac{Measured\;VANC-True\;VANC\;value\;}{True\;VANC\;value}\right)x\;100\%\right)$$

Equation 1: Relative error calculation used for all non-anchor points

where ‘True VANC' represents the known concentration of vancomycin-spiked samples prepared by precise weight-to-volume dilution of vancomycin powder in deionized water and then diluted into whole blood, and “Measured VANC” represents the concentration determined from the calibration curve.

Quality control and assay standardization were achieved through a rigorous batch-matching approach designed to compensate for inter-run signal variation. Each test batch was defined by unique combinations of POEGMA-co-GMA polymerization, device fabrication runs, and final assembly efforts. Calibration curves for each batch were generated using vancomycin-spiked whole blood, with duplicate measurements across a concentration range of 0–50 μg/mL in 2-fold serial dilutions. Vancomycin measurements were calculated using a 5-parameter asymmetric sigmoidal curve fit (GraphPad Prism, version 10.2.3) referencing batch-matched calibration curves. This calibration strategy enabled accurate concentration determination while accounting for batch-specific response characteristics.

#### Clinical validation of the VANC-D4 POCTs using Scavenged samples from CHTN

We conducted initial clinical validation through a collaboration with the Southern Division of the Cooperative Human Tissue Network at Duke University (CHTN). CHTN, established in 1987 by the National Cancer Institute’s Cancer Diagnosis Program, facilitates researcher access to human biological specimens through a network of six regional centers.^82^ This infrastructure enabled us to rapidly and cost-effectively obtain well-characterized VANC-positive blood samples while maintaining compliance with Duke University Health Systems Institutional Review Board approved requirements (Pro00109699, PI Ashutosh Chilkoti), as mentioned above.

#### Clinical validation of the VANC-D4 POCT using CRCs and samples collected in the Surgical intensive care unit at DUMC

The second phase of clinical validation assessed VANC-D4 POCT performance when operated by minimally trained users. Working with clinical research coordinators (CRCs) from Duke University Medical Center’s (DUMC’s) infectious disease department, we conducted parallel testing of prospectively collected samples under the same IRB protocol (Pro00109699). CRCs managed the complete testing workflow, including patient identification, informed consent, and sample collection. For each enrolled patient, paired blood samples were collected via standard phlebotomy: one sample was sent for routine vancomycin testing at DCAL, and another was collected in EDTA-coated tubes for measurement by the VANC-D4 POCT. Samples for POCT testing were maintained at 4°C until 30 min prior to analysis, which was performed by CRCs in the 1K laboratory at DUMC. The study design enabled direct comparison between CRC-operated POCTs and standard laboratory testing while simulating real-world clinical implementation conditions. Quantitative feedback (Figure S8) was gathered from the VANC-D4 POCT User Survey (Appendix A).

### Quantification and statistical analysis

All statistical analysis was performed using GraphPad Prism software version 10.4.1 (GraphPad Software Inc.). Assay precision was quantified as relative error (Equation 1) and reported for each non-anchor concentration point. Accuracy of the VANC-D4 point-of-care test relative to the DCAL reference standard was assessed by linear regression and reported as the coefficient of determination (R^2^). Bland-Altman analysis was used to evaluate agreement between VANC-D4 point-of-care test and DCAL measurements, with bias reported as the mean difference and precision as the standard deviation of differences (reported values: bias = −0.2056 μg/mL; standard deviation = 3.275 μg/mL; 95% limits of agreement = −6.625 to 6.214 μg/mL; *n* = 62 measurements from 31 samples). Calibration curves were generated using a 5-parameter asymmetric sigmoidal curve fit. The exact value of n for each experiment (number of samples, number of replicate runs, or number of devices) is specified in the figure legends and the Results section. No formal statistical method was used to estimate sample size for this pilot study. Data were not tested for normality prior to analysis given the small sample sizes in the clinical validation stages. No data were excluded from analysis except one cassette failure (patient 2, sample run 2, CRC-led Stage 2 test), which was excluded due to a device malfunction rather than on statistical grounds.

## Supplemental information

Document S1. Figures S1–S8 and Tables S1 and S2

Data S1. File containing raw data using for figures and tables in the main text and file S1

Table S3. Age, race/ethnicity, and sex data of blood tested during VANC-D4 POCT development, related to Figures 1–5
