# Supplementary material for: A rapid point-of-care test for vancomycin monitoring at the clinical bedside
Source: iScience. 2026 Jul 9;29(8):116064. doi: 10.1016/j.isci.2026.116064 (PMC13380757; doi:10.1016/j.isci.2026.116064)
Supplement: Document S1. Figures S1–S8 and Tables S1 and S2 [file mmc1.pdf]

iScience, Volume *29*

Supplemental information

A rapid point-of-care test for vancomycin monitoring at the clinical bedside

Damon T. Burrow, David S. Kinnamon, Jason Liu, Jacob T. Heggestad, Simone Wall, Brooke E. Silverstein, Suhail K. Mithani, Vijay Krishnamoorthy, Richard Drew, Daniel Y. Joh, Angus Hucknall, and Ashutosh Chilkoti

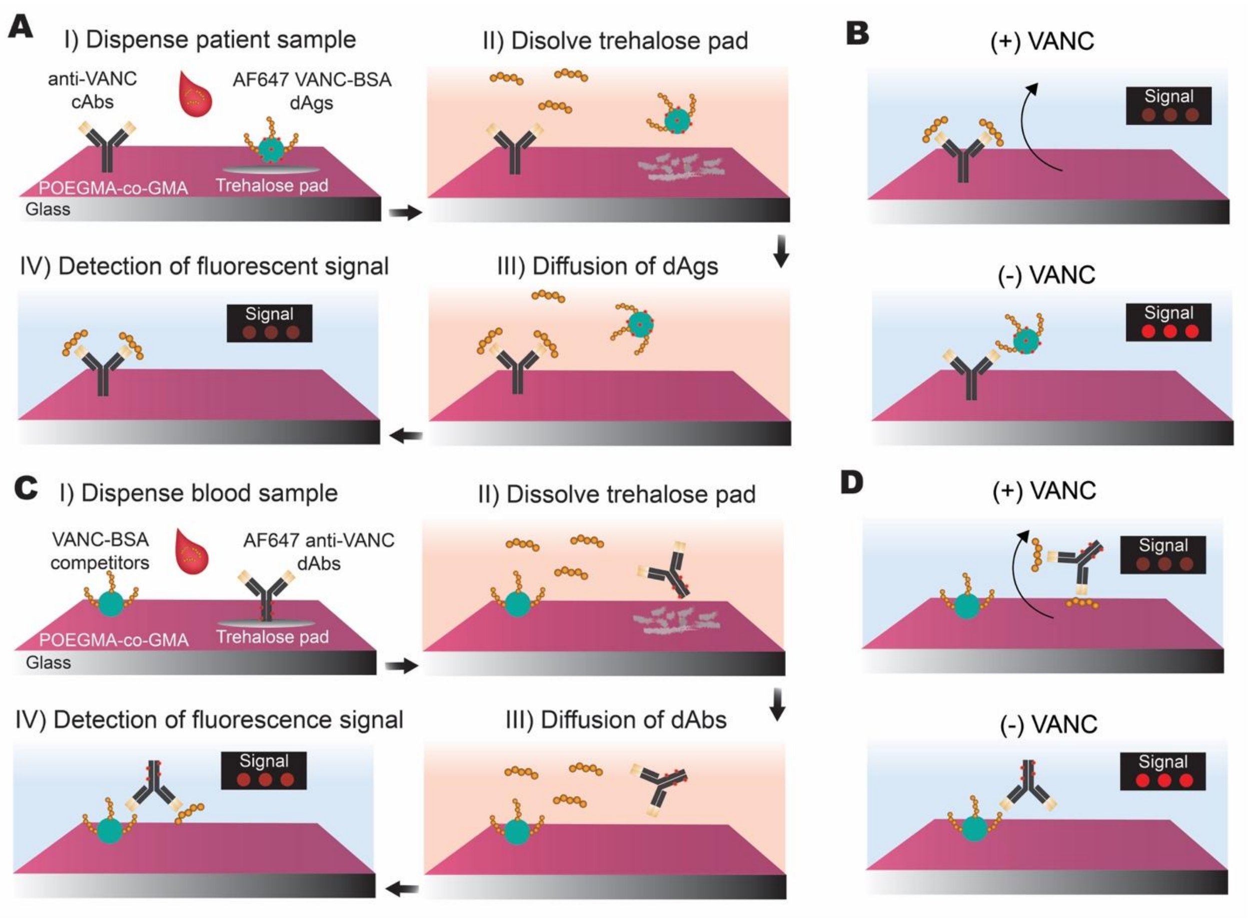

Figure S1: VANC-D4 assay formats, Related to Figure 1, A) Steps of the VANC-D4 capture antibody "cAb" format built upon POEGMA-co-GMA coated glass slides. Here, anti-vancomycin antibodies are immobilized while fluorescently labelled VANC-BSA "detection" competitive antigen (dAgs) are printed onto excipient pads. Step I: Dispensing of the blood sample. Step II: Dissolving of trehalose excipient pads. Step III: Diffusion of dAgs in solution. Step IV: Detection of bound dAgs to immobilized cAbs. B) Representative schematic for the VANC-D4 cAb format response in the presence of drug (top) and absence of drug (bottom). C) Steps of the VANC-D4 capture antibody "dAb" format built upon POEGMA-co-GMA coated glass slides. In this format, VANC-BSA competitive antigen are immobilized while fluorescently labelled anti-vancomycin antibodies (dAbs) are printed onto excipient pads. The D4 steps follow the same as above where - Step I: Dispensing of the blood sample. Step II: Dissolving of trehalose excipient pads. Step III: Diffusion of dAbs in solution. Step IV: Detection of bound dAbs to immobilized cAgs. D) Representative images of the VANC-D4 dAb format response to the presence (top) and absence (bottom) of vancomycin.

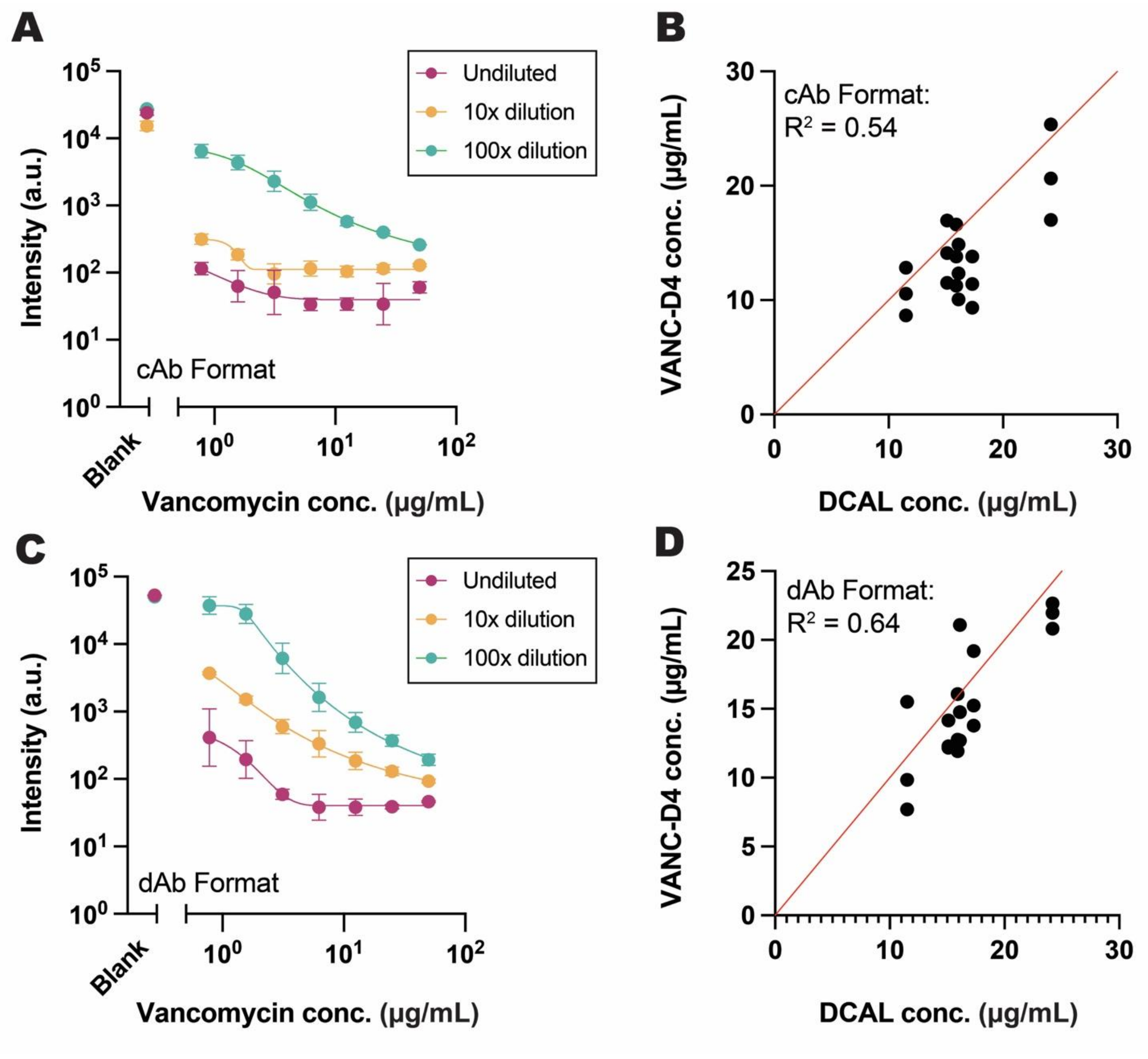

Figure S2: Initial performance comparison for the VANC-D4 cAb and dAb assays, Related to Figure 1, A) Dose-response curves using vancomycin-spiked blood in the cAb format of the VANC-D4 assay. Serial dilutions were created using undiluted (fuchsia), 10-fold diluted (orange) and 100-fold diluted blood (teal). Concentrations are reported for all curves prior to dilution. Data are represented as mean +/- SD for n=3 replicates. B) Comparison of the VANC-D4 cAb format assay to the gold standard test at DCAL using 6 patient samples run in triplicate and plotted individually. X-axis values are taken directly from measurements reported by DCAL and y-axis values are measured directly on the VANC-D4 in the cAb format with a 2.5x scaling factor applied. This 2.5x scaling factor was used only in the cAb format to more closely align the absolute measurements of the VANC-D4 to measurements at DCAL. C) Dose-response curves using vancomycin-spiked blood in the dAb format on the VANC-D4 assay, represented as mean +/- SD for n=3 replicates. D) Comparison of the VANC-D4 dAb format assay to the gold standard test at DCAL using the same 6 patient samples as in B) run in triplicate and plotted individually. No scaling factor was required for dAb format results.

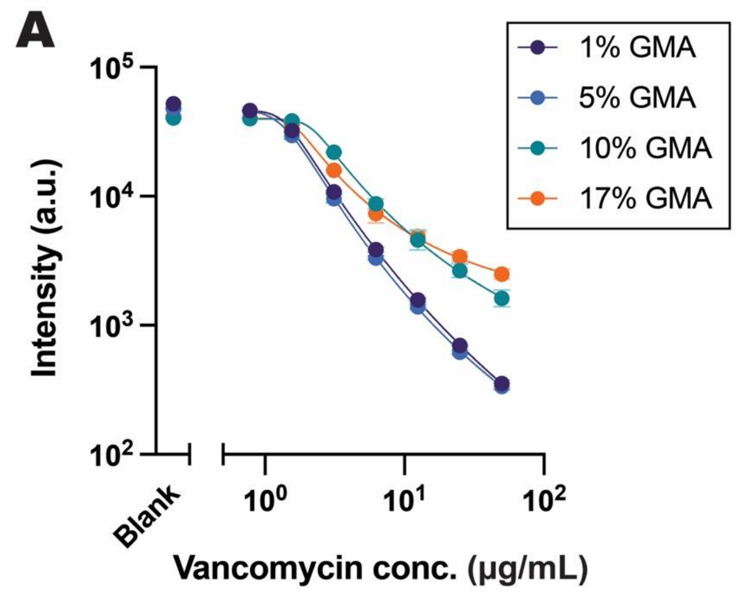

Figure S3: VANC-D4 dose-response curves on POEGMA-co-GMA slides with increasing %GMA, Related to Figure 2, Data for all curves are represented as mean +/- SD, n=3.

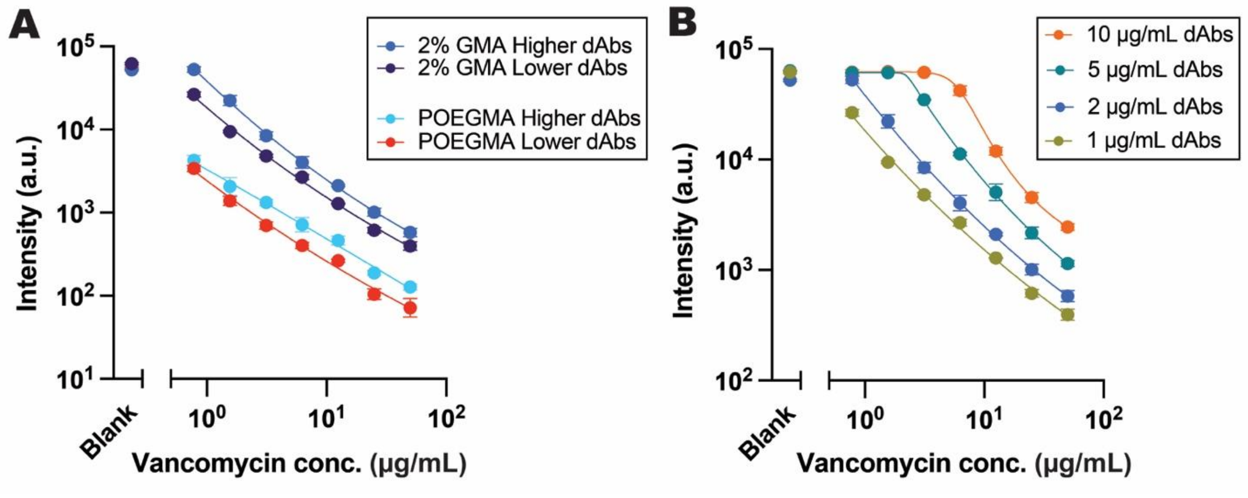

Figure S4: Effect of amount of printed dAb on performance of the VANC-D4 assay in the dAb format, Related to Figure 2, A) Dose-response curves showing tunability of the VANC-D4 for different dAb concentrations on POEGMA-only and POEGMA-co-GMA brushes with 2% GMA. B) Vancomycin dose-response curves on 2% POEGMA-co-GMA brushes at 1 (gold), 2 (blue), 5 (green), and 10 (orange) μg/mL of dAb. Data for S4A-B are plotted in triplicate (n=3) as mean +/- SD.

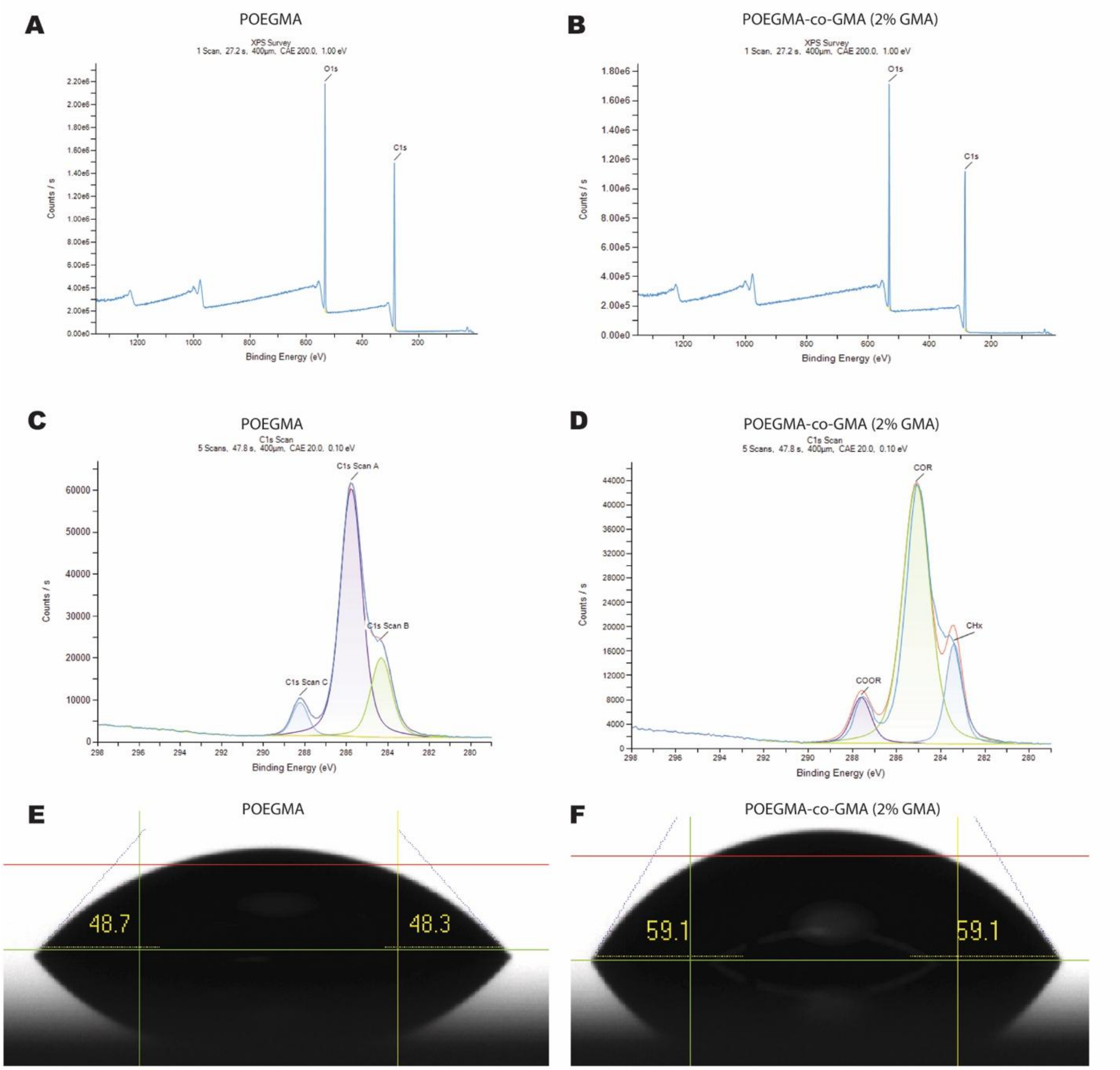

Figure S5: X-ray photoelectron spectroscopy (XPS) and contact angle goniometry characterization of POEGMA and POEGMA-co-GMA brushes, Related to Figure 2, A) XPS survey scan of POEGMA (0% GMA) coated glass slides; and B) POEGMA-co-GMA with 2% GMA coated glass slides. C) XPS C_{1s} scan of glass slides coated with POEGMA brush; and D) POEGMA-co-GMA 2% brush. E) Contact angle goniometer images with measurements using deionized water on POEGMA coated glass slide; and F) on POEGMA-co-GMA 2% coated glass slide.

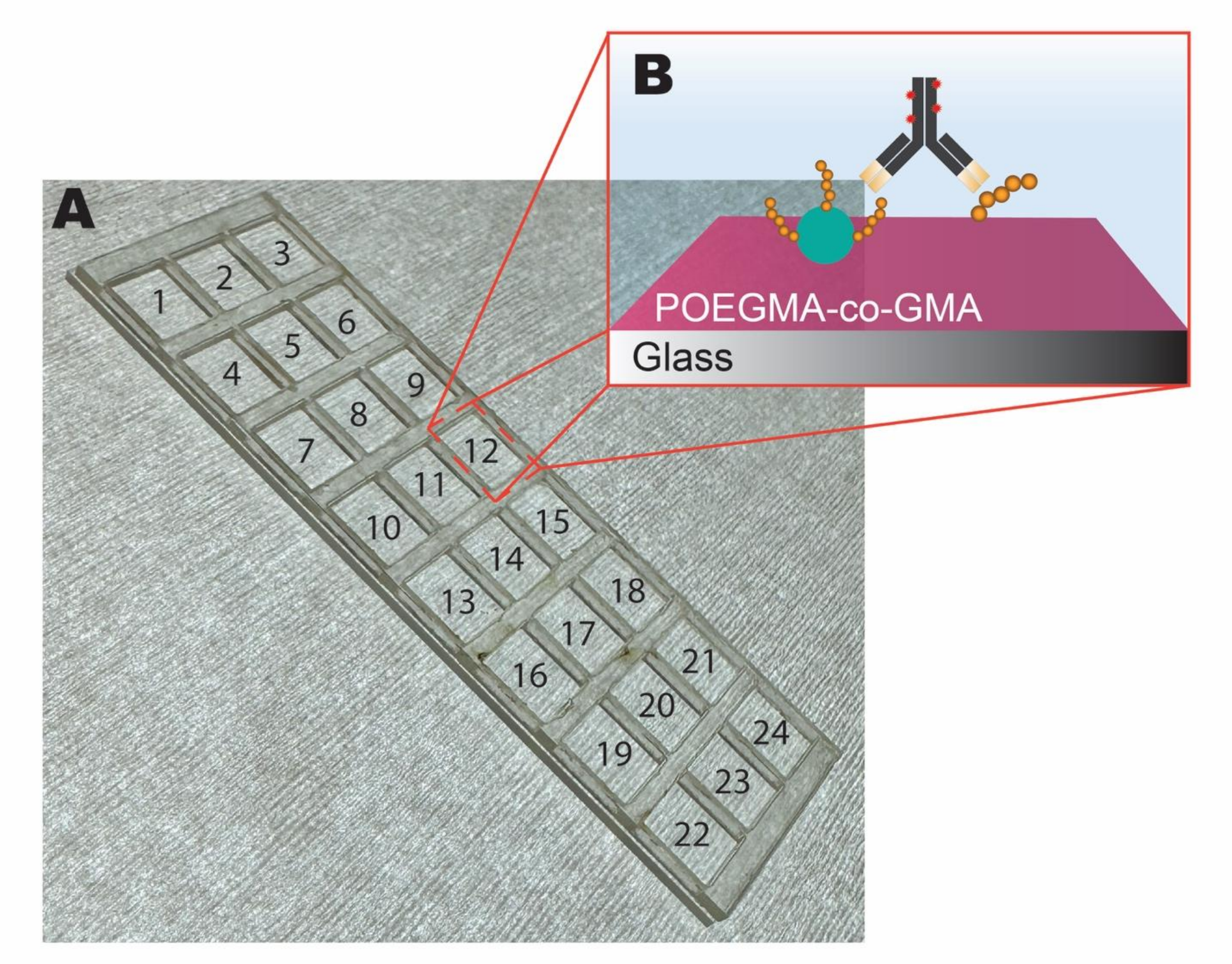

Figure S6: VANC-D4 open-format assay, Related to Figure 1 and Figure 2, A) Image of the open-format VANC-D4 with 24 individual assay wells. B) Expanded schematic showing the VANC-D4 architecture in each well.

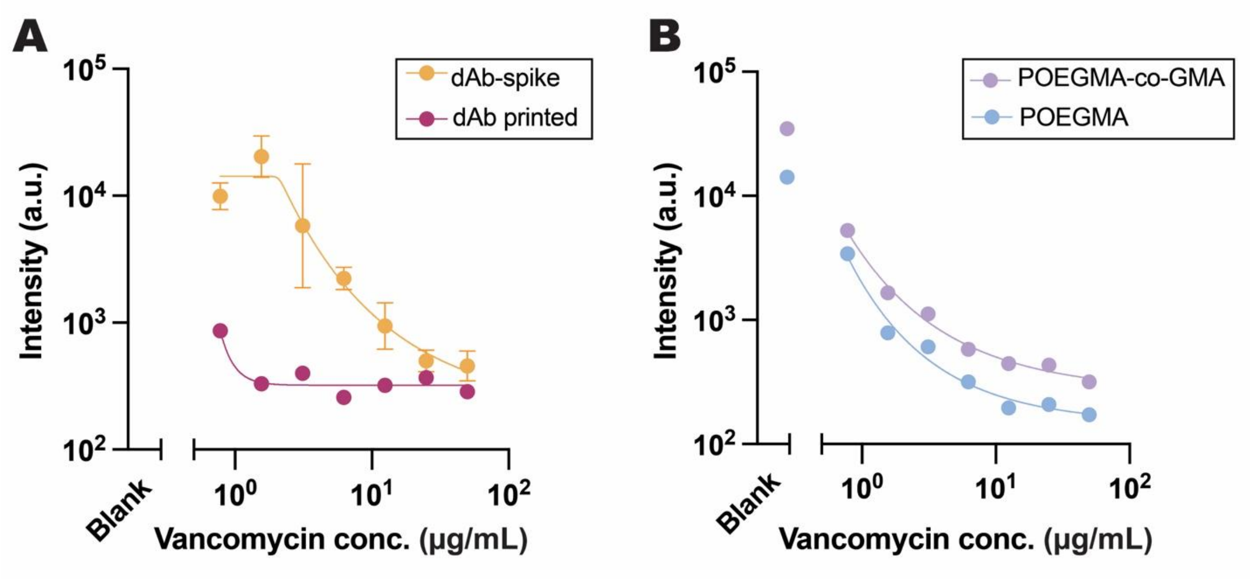

Figure S7: VANC-D4 POCT dAb spike v. dAbs printed on cassette, Related to Figure 4, A) Dose-response curves for assays where the dAbs were printed on the cassette or dAbs were spiked in the VANC-D4 POCT. B) Comparison using POEGMA-only and POEGMA-co-GMA brushes in the VANC-D4 POCT using the dAb-spike approach. Data shown n=2, mean +/- SD.

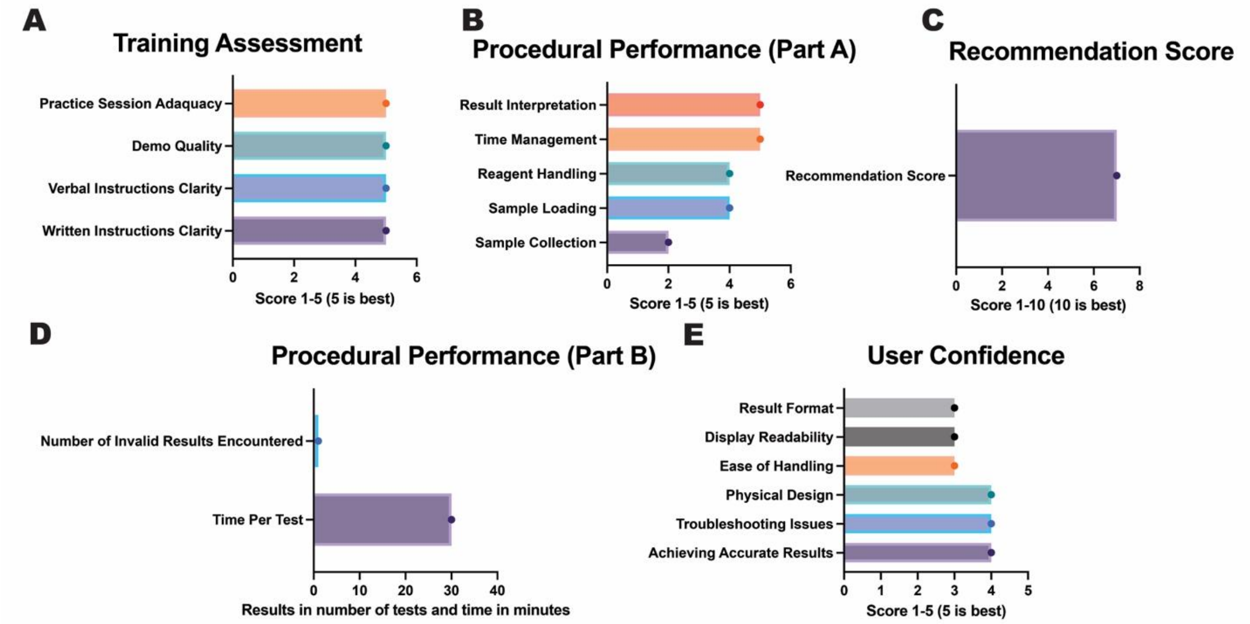

Figure S8: VANC-D4 POCT response from CRC user survey, Related to Figure 4, A) Response to training assessment questions from the primary CRC users at Duke University Medical Center. B) Overall recommendation score from the CRCs. C) Procedural performance, part B, answer from the CRCs. D) Procedural performance, part B, answer from the CRCs. E) User confidence scores from the CRCs.

Table S1: RE values for VANC-D4 assays on POEGMA and POEGMA-co-GMA coated slides with differing GMA percent and dAb amounts, Related to Figure 2.

POEGMA 1 µg/mL dAbs	
VANC conc. (µg/mL)	relative error percent (RE%)
25	24%
12.5	21%
6.25	8%
3.125	9%
1.5625	11%
POEGMA 2 µg/mL dAbs	
VANC conc. (µg/mL)	relative error percent (RE%)
25	20%
12.5	15%
6.25	18%
3.125	8%
1.5625	29%
POEGMA-co-GMA (2% GMA) 1 µg/mL dAbs	
VANC conc. (µg/mL)	relative error percent (RE%)
25	12%
12.5	4%
6.25	10%
3.125	4%
1.5625	11%
POEGMA-co-GMA (2% GMA) 2 µg/mL dAbs	
VANC conc. (µg/mL)	relative error percent (RE%)
25	11%
12.5	6%
6.25	12%
3.125	7%
1.5625	9%
POEGMA-co-GMA (2% GMA) 5 µg/mL dAbs	
VANC conc. (µg/mL)	relative error percent (RE%)
25	8%
12.5	11%
6.25	4%
3.125	Concentration value off curve
1.5625	Concentration value off curve
POEGMA-co-GMA (2% GMA) 10 µg/mL dAbs	
VANC conc. (µg/mL)	relative error percent (RE%)

25	7%
12.5	3%
6.25	6%
3.125	Concentration value off curve
1.5625	Concentration value off curve

Table S2: Patients samples used for the clinical comparison of the VANC-D4 POCT to DCAL, Related to Figure 5.

Sample Number	*DCAL measured values (µg/mL)*	*VANC-D4 POCT measured values (µg/mL)*	
SD-24-330	11.5	10.10	9.91
SD-24-331	11.3	11.95	10.71
SD-24-332	27.7	36.09	42.90
SD-24-333	14.4	11.17	11.15
SD-24-334	9.9	7.49	7.39
SD-24-335	19.3	23.64	18.58
SD-24-336	8.2	10.07	7.84
SD-24-337	2*	1.12	1.38
SD-24-338	7	7.59	5.47
SD-24-339	9.5	9.43	9.72
SD-24-340	19.4	23.48	15.50
SD-24-341	24.3	23.64	30.66
SD-24-342	12.4	11.31	11.75
SD-24-343	9.2	8.03	8.67
SD-24-344	9.6	8.50	8.47
SD-24-345	9.5	10.73	8.66
SD-24-346	20	18.58	21.52
SD-24-347	7.5	7.88	7.08
SD-24-348	13.2	11.35	11.47
SD-24-349	19.4	15.77	15.58
SD-24-351	17.8	15.16	13.11
SD-24-352	9.3	10.61	12.44
SD-24-353	9.7	11.09	10.60
SD-24-354	6.4	9.97	5.97
SD-24-355	14.7	10.13	10.19
SD-24-357	15.8	14.28	12.41
9165-1	6.2	5.25	5.09
9165-2	14.6	14.49	**cassette failure
9165-3	8.6	6.32	7.00

9165-4	13.5	12.21	19.39
9165-5	6	6.16	4.49
